# SEOM clinical guidelines for the treatment of renal cell carcinoma

**DOI:** 10.1007/s12094-014-1219-1

**Published:** 2014-10-02

**Authors:** J. Bellmunt, J. Puente, J. Garcia de Muro, N. Lainez, C. Rodríguez, I. Duran

**Affiliations:** 1 Medical Oncology, Dana Farber Cancer Center/Harvard Medical School, Boston US and Hospital del Mar-IMIM, Barcelona, Spain; 2 Medical Oncology Department, Hospital Clínico San Carlos, Madrid, Spain; 3 Medical Oncology Department, Hospital Universitario Virgen del Rocío, Seville, Spain; 4 Medical Oncology Department, Institut Català d’Oncologia L’Hospitalet, Barcelona, Spain; 5 Medical Oncology Department, Complejo Hospitalario de Navarra, Pamplona, Spain; 6 Medical Oncology Department, Hospital Universitario de Salamanca, Salamanca, Spain

**Keywords:** Renal cell carcinoma, SEOM, Guidelines

## Abstract

The purpose of this article was to provide updated recommendations for the diagnosis and treatment of renal cell carcinoma. Pathological confirmation is mandatory before treatment with ablative or focal therapies before any type of systemic therapy. Renal cell cancer should be staged according to the TNM classification system. A laparoscopic nephron-sparing surgery should be the approach for tumors <4 cm if technically feasible. Otherwise, radical (or partial in selected cases) nephrectomy is the treatment of choice, with lymph node dissection only performed in patients with clinically detected lymph node involvement. Some retrospective evidence for a cytoreductive nephrectomy in the postimmunotherapy era suggests a benefit in patients with good or intermediate risk or for patients with a symptomatic primary lesion. Adjuvant treatment with chemotherapy or with targeted agents is not recommended and studies are ongoing today. Patients with metastatic disease should be staged by computed tomography scans of the chest, abdomen and pelvis. The efficacy of sunitinib, bevacizumab plus interferon-α, and pazopanib is well established in patients with good and intermediate risk as well for temsirolimus in poor-risk patients. These four agents are considered standard of care in first-line treatment. Sorafenib, axitinib and everolimus are standard of care in second line in different settings based on their benefit in PFS. Besides some benefit described for IL-2 in highly selected patients in first line, there is a promising and emerging role for the new immunotherapeutic approaches in metastatic renal cell carcinoma.

## Incidence and survival rates

Renal cancer is the 12th most common malignancy worldwide (338,000 new cases diagnosed in 2012) with 6,474 new cases diagnosed in 2012 in Spain [1]. It occurs more often in men than in woman (age-standardized ratios for both incidence and mortality are 50 % higher in man compared with woman). Worldwide incidence of all stages has increased in recent years, 2 % yearly, and was responsible for over 143,469 deaths in 2012 [2].

## Risk factors

Approximately 75 % of renal cancers are diagnosed over the age of 60, with a plateau reached around 70–75 years of age [2].

Smoking is a well-established risk factor for renal cancer with a meta-analysis reporting a clear difference between smokers and non-smokers and also a dose-dependent risk in number of cigarettes smoked [3]. Smoking cessation for more than 10 years may reduce the risk of RCC [4]. Obesity has also been established as a risk factor for RCC. A meta-analysis provided evidence for an association between body mass index and risk of RCC [5]. Several studies have demonstrated an inverse relationship between physical activity and RCC risk. Several cohort studies have reported an association with long-term hypertension and risk of RCC [6].

Approximately 2–3 % of RCCs are familial or hereditary [7] with a twofold increase incidence in a first-degree relative. Each histological subtype has a corresponding hereditary component caused by distinct genetic alteration. The most common hereditary syndrome for clear cell RCC is the von Hippel–Lindau (VHL) syndrome. Hereditary papillary RCC is associated exclusively with type 1 papillary RCC and does not present with manifestations in other organs. The familial leiomyomatosis and RCC syndrome have been correlated with mutations in the fumarate hydratase gene (FH) and patients present with type 2 papillary RCC. Lastly, in the Birt–Hogg–Dubé (BHD) syndrome, germ line mutations in the homonymous tumor suppressor folliculin gene are characteristic and patients present typically with chromophobe RCC, oncocytomas or hybrid tumors.

## Pathological diagnosis/molecular biology

More than 50 % of RCCs are currently detected incidentally when imaging is being performed for some other reasons. However, still a large number of patients with RCC present with symptoms, such as flank pain, gross hematuria and palpable abdominal mass. This is considered the “classical triad” for diagnosis, nowadays infrequently seen together. Metastatic symptoms such as bone pain, dyspnea, cough or paraneoplastic syndromes such as hypercalcemia, unexplained fever, erythrocytosis or wasting syndromes are occasionally seen, being this the reason the name RCC as “the internist tumor”.

A core biopsy can provide the confirmation of malignancy with the specific histological type with high sensitivity and specificity. Biopsy is mandatory especially before the treatment with ablative or focal therapies [3, B]. It is also mandatory in patients with metastatic disease before starting any type of systemic treatment [3, B]. The final histopathological diagnosis, classification, grading and evaluation of prognostic factors are based on the nephrectomy specimen when available.

Different histological subtypes with specific genetic alterations have been identified with similar genetic alterations seen in the familial forms.

### Clear cell renal cell carcinoma

Clear cell RCC (ccRCC) is the most common subtype of renal cancer, accounting for 75 % of all primary kidney tumors. These tumors have clear cytoplasm secondary to deposition of lipids and glycogen. Clear cell tumors are commonly hypervascular and can show coagulative tumor necrosis. The most characteristic feature seen in ccRCC is the inactivation of von Hippel–Lindau (VHL) tumor suppressor gene [8] reported in up to 75 %. Other commonly observed cytogenetic alterations in ccRCC include losses at 3p (90 %) 14q, 8p and 9p and gains at 12q and 5q [9]. Inactivation of several histone-modifying genes has been described, such as SETD2 (10–12 %), PBRM1 (40 %) and BAP1 (10 %) with suggested prognostic implications [10].

### Papillary renal cell carcinoma

Papillary RCC types occur in 10–15 % of cases. Multifocal and synchronous bilateral cases are observed in 10 % of papillary RCC. Two subtypes are described: papillary type 1 and type 2.

Germ line met proto-oncogene (MET) and FH alterations are observed in the hereditary form of papillary 1 and in the hereditary leiomyomatosis (type 2), respectively. However, these genetic abnormalities are not frequently observed in the sporadic forms. Cytogenetic alterations in papillary RCC type 1 include gains of chromosomes 7, 8q, 12q, 16p, 17, 20 and loss of 9p. Papillary type 2 tumors gain 8q, lose 1p and 9p [11].

There are conflicting data on the differences/similarities in clinical behavior between papillary RCC type 1 and type 2. Nevertheless, the former appears to be associated with fewer aggressive features than the latter, including a lower stage and grade, as well as longer 5-year survival (~89–94 % versus 55–74 %) [12].

### Chromophobe renal cell carcinoma

Chromophobe RCC occurs in 5 % of cases. This subtype has a less aggressive phenotype when compared with the other histological subtypes. Whether or not patients with chromophobe RCC have a better survival outcome than those with other histological subtypes is unclear. Chromophobe histology is present in 30 % of renal tumors seen in the hereditary Birt–Hogg–Dubé (BHD) syndrome [13]. This disease is associated with mutations in the BHD gene whose product is folliculin. Chromophobe tumors frequently have copy number alterations at chromosome 1, 2, 6, 10, 13 and 17. Strong staining for cell membrane-bound KIT protein has consistently been shown in chromophobe RCC tumors.

### Sarcomatoid transformation

Sarcomatoid transformation is present in 5 % of RCC. Sarcomatoid components can occur in all histological subtypes of RCC and do not in themselves represent a distinct histological entity. It has been suggested that a cutoff of 30 % sarcomatoid features in the primary tumor may be useful in predicting systemic sarcomatoid histology. TP53 alterations occur in this dedifferentiation process [14].

### Collecting duct renal cell carcinoma

Collecting duct RCCs account for less than 0.5 % of RCC. These tumors arise from the medullary distal nephron or Bellini ducts. Bellini ducts tumors are an aggressive histological subtype, and most patients have metastases at presentation. Cytogenetic abnormalities include losses at 1p, 8p, 9p and 16p and gains at 13q [15].

### Other types

Other less common subtypes include renal translocation carcinomas.

This rare entity of renal translocation carcinomas was first observed in children and young adults but has also been reported in adults. This tumor is characterized by the translocation of Xp11.2, with the gene fusions involving the TFE3 transcription factor gene or TFEB. More than 10 additional histological subtypes have been defined which occur rarely. These include the unclassified RCC, medullary, multilocular cystic RCC, mucinous tubular and spindle cell carcinoma and carcinoma associated with end-stage renal disease. The last WHO classification should be used to classify histology in RCC.

## Staging of RCC: prognostic models for risk assessment

### Staging of RCC

The staging of renal cell carcinoma (RCC) should be done according to the TNM classification system (version 2009) [16] (Tables 1 and 2).

**Table 1:** AJCC TNM staging for RCC (7th ed.)

Primary tumor (T)	
T0	No evidence of primary tumor
T1	Tumor <7 cm in diameter and limited to kidney
T1a	Tumor ≤4 cm in diameter and limited to kidney
T1b	Tumor ≤4 cm but <7 cm and limited to kidney
T2	Tumor >7 cm in diameter and limited to kidney
T2a	Tumor >7 cm but <10 cm in diameter and limited to the kidney
T2b	Tumor >10 cm and limited to the kidney
T3	Tumor extends into major veins or perinephric tissues, but not beyond Gerota’s fascia
T3a	Tumor directly invades perinephric tissues, but not beyond Gerota’s fascia
T3b	Tumor grossly extends into vena cava below the diaphragm
T3c	Tumor grossly extends into vena cava above diaphragm or invades wall of vena cava
T4	Tumor invades beyond Gerota’s fascia (including contiguous ipsilateral adrenal gland)
Regional lymph nodes (N)^a^	
N0	No regional lymph node metastasis
N1	Metastasis in regional lymph node (s)
Distant metastasis (M)	
M0	No distant metastasis
M1	Distant metastasis

^a^N classification not affected by laterality

**Table 2:** Stage grouping for RCC Based on AJCC TNM stage and survival

Stage	T	N	M	5-year OS (%)
Stage I	T1	N0	M0	81
Stage II	T2	N0	M0	74
Stage III	T1, T2	N1	M0	53
	T3	Any N	M0	
Stage IV	T4	Any N	M0	10
	Any T	Any N	M1	

### Evaluation of local disease

#### Computed tomography (CT) scan

The abdominal CT scan represents the gold standard in the staging of RCC and must be performed with and without intravenous [3, A] contrast and including images from the nephrographic phase. A change in 15 or more Hounsfield units before and after the contrast administration will be diagnostic of enhancement and suggesting malignancy. Abdominal CT imaging will provide relevant information for staging including: (1) degree of extension of primary tumor; (2) involvement of vasculature; (3) regional lymph nodes status; (4) adrenal gland and liver involvement; and (5) morphology and function of the contralateral kidney.

#### Magnetic resonance imaging (MRI)

Abdominal MRI is not performed routinely in the staging of RCC.

Indications of staging using MRI include: (1) allergy to CT iv contrast or pregnancy; (2) indeterminate results of CT regarding enhancement of complex renal masses; and (3) investigation of venous involvement when poor definition of inferior vena cava tumor thrombus in CT scan or investigation of locally advanced disease. Despite a high accuracy of both CT and MRI in RCC diagnosis, these tests are not able to reliably distinguish oncocytoma and fat-free angiomyolipoma from RCC [17].

#### Other imaging tests

##### Vascular imaging studies

Studies such as arteriography/venacavography will be utilized only in selected cases.

##### Radioisotope renography

Renal function evaluation studies should be considered when contemplating nephron-sparing strategies or if any sign of impaired renal function is present pre-surgery.

##### Positron emission tomography (PET)

There is no defined role for PET in diagnosis or follow-up of RCC. It should be considered investigational [1, B].

### Evaluation of advanced disease

#### Chest imaging evaluation

In addition to the abdominal CT, the most accurate imaging test for chest staging is the chest CT that should be considered at initial staging [3, A]. An alternative is the chest X-ray [18].

#### Bone or brain studies

Outside a clinical trial, imaging studies of bone or brain should be performed only in the presence of symptoms or specific laboratory abnormalities [3, A] [19].

### Risk assessment

Different variables including anatomical factors (i.e., size of the tumor, renal capsule invasion, venous invasion, adrenal and lymph node involvement), histological factors (i.e., RCC subtype, tumor necrosis and Fuhrman grade) and clinical factors (i.e., PS, cachexia, anemia, platelet count or local symptoms) are incorporated in prognostic models both in localized and metastatic disease. The UISS, SSIGN and the postoperative Karakiewicz’s nomogram are the most widely used prognostic models in localized disease [20–22].

In the advanced setting, the MSKCC and the Heng classification are the two most spread prognostic models although others have also been developed [23, 24] (see Table 3).

**Table 3:** MSKCC or Heng risk criteria

MSKCC risk criteria (prognostic factors for poor OS)
KPS <80
Diagnosis to therapy <1 year
Anemia
Hypercalcemia
Elevated LDH
0 factors: favorable risk
1–2 factors: intermediate risk
≥3 factors: poor risk

Heng risk criteria for VEGF-targeted therapy [prognostic factors for poor overall survival (OS)]
KPS <80
Diagnosis to therapy <1 year
Anemia
Hypercalcemia
Neutrophilia
Thrombocytosis
0 factors: favorable risk
1–2 factors: intermediate risk
≥3 factors: poor risk

While the MSKCC or Heng criteria should be routinely utilized in the treatment decision process of the patient with advanced RCC, the prognostic models in the localized setting remain investigational until data of prospective ongoing adjuvant studies are available.

## Management of localized/resectable disease

### Local disease

Surgery (nephrectomy) is the best approach with curative intention for localized RCC. Different techniques can be performed based on the extension of the resection (nephron sparing [NSS] or radical [RN]) or the approach (open vs. laparoscopic). For RCC tumors <4 cm, RN has been associated with increased mortality when compared with NSS. Therefore, RN is not recommended in this context unless a NSS is not technically feasible [25]. These outcomes have not been replicated in RCC tumors of 4–7 cm where partial and radical nephrectomy achieved similar cancer-specific survival and overall survival [26].

Regarding the approach, when compared with radical open surgery (ROS), radical laparoscopic nephrectomy (RLN) seems to have less surgical related complications although no differences in oncological outcomes have been demonstrated. Therefore, when a radical approach is recommended, RLN should be prioritized [27].

When analyzed by stage, RCC tumors stage 1 (T1) should be treated with a laparoscopic NSS if technically feasible. Stage 2 (T2) tumors should be handled with RLN. Stage III (T3, T4) tumors should be treated with open radical nephrectomy. Neither extended lymph node dissection nor adrenalectomy has shown added survival benefit and should not be performed routinely unless there is radiological or intraoperatory evidence of node involvement [28, 29].

Active surveillance (AS) (described as an initial watching of tumor size by successive abdominal imaging tests with deferred intervention reserved for those tumors that show clinical progression during follow-up) is an acceptable option in elderly and comorbid patients with small renal masses (<4 cm) detected incidentally as these masses tend to have a relatively low RCC-specific mortality [30].

Adjuvant treatment is not recommended in patients at high risk of relapse. Adjuvant trials with targeted therapies are ongoing or have completed accrual, and results are not yet available. Neo-adjuvant approaches are investigational and are not recommended in daily practice.

## Management of advanced metastatic disease: first-line, second-line and therapeutic sequences––therapeutic algorithm

### Role of surgery

In the era of cytokines, cytoreductive nephrectomy before systemic therapy was shown to provide a survival benefit in patients with good PS [I, A].

In the era of targeted therapies, we have retrospective evidence that cytoreductive nephrectomy can be of benefit in patients with good or intermediate risk or for patients with a symptomatic primary lesion [4, B]. Prospective trials are ongoing.

Metastasectomy can be considered in selected patients with solitary or limited number (≤4) of lung metastases and in solitary resectable metastases in other locations with long metachronous disease-free interval. It can also be considered in selected patients with stable responses to targeted therapies [31].

### First-line treatment

The efficacy of sunitinib, bevacizumab plus interferon (IFN)-α [1, A], pazopanib [1, B] and temsirolimus [1, A] as first-line therapy was compared with either IFN-α or placebo in separate randomized phase III trials [32–36]. Results showed that each of these targeted agents was superior to IFN-α in prolonging progression-free survival or overall survival times, or both. The majority of the patients in the sunitinib, pazopanib and bevacizumab plus IFN-α trials were in the favorable or intermediate MSKCC risk groups, and benefits relative to IFN-α were observed across groups. In the temsirolimus trial, all patients were classified by similar criteria as having a poor prognosis, which was equivalent to 74 % of patients being classified in the MSKCC poor-risk group and 26 % of patients being classified in the MSKCC intermediate-risk group.

The results of these trials prompted many changes in first-line therapy recommendations. Although Interferon plus bevacizumab have demonstrated similar impact than oral agents as a first-line therapy for metastatic RCC, oral monotherapy with a TK inhibitor has become the “de facto” standard of care in this situation.

Sunitinib has been the first agent showing high activity in first line. To date, three phase III trials have compared two different TKIs. After the phase III and the non-inferiority COMPARZ trials (median PFS 8.4 vs. 9.5 months with pazopanib and sunitinib, respectively), pazopanib has become an alternative option to sunitinib with some differences in toxicity profile, as first-line therapy for advanced or metastatic RCC [37]. In addition, two randomized trials have recently compared sorafenib with either tivozanib or axitinib. Tivozanib has become the first TKI that showed a benefit in terms of PFS in a phase III trial over another TKI, but was not approved because of its lack of impact in survival [38]. On the other hand, axitinib did not reach the pre-established target of the study, and the trial was prematurely discontinued [39].

#### Recommendations (clear cell histology)

1. Sunitinib and pazopanib are best first-line treatment alternatives in metastatic RCC patients with good and intermediate risk (level of evidence: I; grade of recommendation: A and B, respectively).
2. Bevacizumab combined with interferon is also an option, although it has been less used in favor of more convenient use of oral therapies (level of evidence: I; grade of recommendation: A).
3. In patients with poor risk features, temsirolimus constitutes the first-line therapy (level of evidence: I; grade of recommendation: A), although sunitinib may also be an option for these patients (level of evidence: II; grade of recommendation: B).
4. At this moment, first-line treatment with immunotherapy should not be recommended for patients with metastatic RCC (level of evidence: I; grade of recommendation: A).
5. In highly selected fit patient population, high dose IL-2 could be considered (level of evidence: I; grade of recommendation: D).

### Second-line treatment and therapeutic sequences

Based on a phase III study, the TKI sorafenib is approved for patients with advanced RCC, in whom cytokine therapy has failed or was not indicated [I, A] [40]. Sorafenib almost doubled PFS in second-line therapy compared to placebo (2.8 vs. 5.5 months). With axitinib, a second TKI has recently become available for the second-line therapy of patients after failure of first-line therapy with sunitinib or a cytokine [I, A]. In the AXIS study [41], PFS was increased by 2 months (6.7 vs. 4.7 months) with axitinib compared to sorafenib as reference [I, B]. Moreover, in second-line therapy, with everolimus an mTOR inhibitor can be used to treat these patients [II, A]. In the phase III RECORD-1 study, this agent was compared with placebo in patients after failure of at least 1 anti-VEGF therapy and increased median PFS (1.9 vs. 4.9 months) [42].

A phase III trial comparing an mTOR inhibitor, temsirolimus, with a second TKI, sorafenib, following progression on first-line sunitinib has been reported [43]. No differences in PFS were found (4.2 vs. 3.9 months). However, a significant advantage in OS in favor of sorafenib was observed (16.6 vs. 12.2 months; *p* = 0.014). The results of this study could be related to subsequent treatments administered, but unfortunately, these data are lacking. However, this finding and other retrospective studies [44] give more support to the sequence TKI after TKI. Nevertheless, mTOR inhibitors are also active after a second TKI and could be a good alternative in patients with severe toxicity on a previous TKI. Recently, a randomized phase II study that compared the sequence of first-line sunitinib followed by second-line everolimus with the reverse sequence showed a superiority in terms of PFS and OS favoring the sunitinib––everolimus sequence [45], suggesting that the order of administration of the agents is not irrelevant.

#### Recommendations

1. After progression to first-line therapy with a TKI, sequential administration of alternative targeting agents should be considered (level of evidence: I; grade of recommendation: A). In this setting, both sequences either administering a second TKI or mTOR inhibitor are active therapeutic alternatives (level of evidence: I, B for everolimus and I, B for axitinib).
2. Axitinib has been shown to be superior to sorafenib in second-line treatment (level of evidence: I; grade of recommendation: A), but sorafenib could be even consider an active option (level of evidence: IV; grade of recommendation: B).
3. Sequential therapy with mTOR inhibitors should be considered in patients who progress after a second TKI (level of evidence: III; grade of recommendation: B) or in those patients who experienced poor tolerance to a first-line TKI (level of evidence: IV; grade of recommendation: B).

### Treatment of metastatic non-clear cell histology

Some studies suggest now that patients with non-clear cell histology may benefit from treatment with sunitinib, sorafenib or temsirolimus [III, B]. The recent communication of ESPN trial (Tannir, N ASCO 2014) confirms that everolimus is not considered today the first option for therapy and still the optimal therapy remains unclear and warrants further study [46] (Table 4).

**Table 4:** Treatment algorithm

Treatment status	Setting	Category I evidence	Category II evidence
Treatment naive (ccRCC)	Good intermediate risk	Sunitinib Bevacizumab/Interferon Pazopanib	Sorafenib High dose IL-2
	Poor risk	Temsirolimus	Sunitinib Sorafenib
Second-line (ccRCC)	Cytokine refractory	Sorafenib Pazopanib Sunitinib Axitinib	
	TKI failure	Everolimus Axitinib	Sorafenib
	Prior mTor inhibitors		Sunitinib
Non-Clear Cell histology			Temsirolimus Everolimus Sunitinib Sorafenib

## Response evaluation and follow-up

### Response evaluation

Currently, response evaluation in patients with advanced RCC is generally accepted to be performed every 8–12 weeks with CT chest–abdomen–pelvis as the method of choice. Response Evaluation Criteria in Solid Tumors (RECIST) is of limited utility in this setting but still remains the standard. Other imaging changes such as those integrated in the modified Choi criteria could be more accurate in correlating with clinical outcomes when using tyrosine kinase inhibitors. Special caution is needed when interpreting moderate size changes with density variations and no new lesions to avoid misinterpretation of progression in patients with advanced RCC [47, 48].

### Follow-up

After definitive local treatment of RCC, there is no consensus about the best follow-up protocol. The most widely accepted approach is stratifying patients based on Fuhrman Grade, TNM Stage, ECOG and type of local treatment, in risk groups. Those patients considered with intermediate or high risk of relapse should be followed more intensively (3 versus 6 months imaging). CT of the chest–abdomen and pelvis is the imaging test of choice although no clear data about the proper timing and/or number of test per year is available (Table 5).

**Table 5:** Levels of evidence and grades of recommendation (adapted from the Infectious Diseases Society of America—United States Public Health Service Grading System)

Levels of evidence	
I	Evidence from at least one large randomised, controlled trial of good methodological quality (low potential for bias) or meta-analyses of well-conducted randomised trials without heterogeneity
II	Small randomised trials or large randomised trials with a suspicion of bias (lower methodological quality) or meta-analyses of such trials or of trials with demonstrated heterogeneity
III	Prospective cohort studies
IV	Retrospective cohort studies or case–control studies
V	Studies without control group, case reports, experts’ opinions
Grades of recommendation	
A	Strong evidence for efficacy with a substantial clinical benefit, strongly recommended
B	Strong or moderate evidence for efficacy but with a limited clinical benefit, generally recommended
C	Insufficient evidence for efficacy or benefit does not outweigh the risk or the disadvantages (adverse events, costs, etc.), optional
D	Moderate evidence against efficacy or for adverse outcome, generally not recommended
E	Strong evidence against efficacy or for adverse outcome, never recommended

## References

[1] Chavan S, Bray F, Lortet-Tieulent J, Goodman M, Jemal A. International variations in bladder cancer incidence and mortality. Eur Urol. 2014;66(1):59–73. 10.1016/j.eururo.2013.10.001 24451595

[2] Chow WH, Devesa SS (2008). Contemporary epidemiology of renal cell cancer. Cancer J.

[3] Hunt JD, van der Hel OL, McMillan GP, Boffetta P, Brennan P (2005). Renal cell carcinoma in relation to cigarette smoking: meta-analysis of 24 studies. Int J Cancer.

[4] Parker AS, Cerhan JR, Janney CA, Lynch CF, Cantor KP (2003). Smoking cessation and renal cell carcinoma. Ann Epidemiol.

[5] Renehan AG, Tyson M, Egger M, Heller RF, Zwahlen M (2008). Body-mass index and incidence of cancer: a systematic review and meta-analysis of prospective observational studies. Lancet.

[6] Weikert S, Boeing H, Pischon T, Weikert C, Olsen A, Tjonneland A (2008). Blood pressure and risk of renal cell carcinoma in the European prospective investigation into cancer and nutrition. Am J Epidemiol.

[7] Coleman JA. Familial and hereditary renal cancer syndromes. Urol Clin North Am. 2008;35(4):563–72. 10.1016/j.ucl.2008.07.014 18992610

[8] Nickerson ML, Jaeger E, Shi Y, Durocher JA, Mahurkar S, Zaridze D (2008). Improved identification of von Hippel–Lindau gene alterations in clear cell renal tumors. Clin Cancer Res.

[9] Network CGAR (2013). Comprehensive molecular characterization of clear cell renal cell carcinoma. Nature.

[10] Brugarolas J (2014). Molecular genetics of clear-cell renal cell carcinoma. J Clin Oncol.

[11] Klatte T, Pantuck AJ, Said JW, Seligson DB, Rao NP, LaRochelle JC (2009). Cytogenetic and molecular tumor profiling for type 1 and type 2 papillary renal cell carcinoma. Clin Cancer Res.

[12] Bellmunt J, Dutcher J (2013). Targeted therapies and the treatment of non-clear cell renal cell carcinoma. Ann Oncol.

[13] Pavlovich CP, Walther MM, Eyler RA, Hewitt SM, Zbar B, Linehan WM (2002). Renal tumors in the Birt–Hogg–Dubé syndrome. Am J Surg Pathol.

[14] Shuch B, Bratslavsky G, Linehan WM, Srinivasan R (2012). Sarcomatoid renal cell carcinoma: a comprehensive review of the biology and current treatment strategies. Oncologist.

[15] Becker F, Junker K, Parr M, Hartmann A, Füssel S, Toma M (2013). Collecting duct carcinomas represent a unique tumor entity based on genetic alterations. PLoS One.

[16] Novara G, Ficarra V, Antonelli A, Artibani W, Bertini R, Carini M (2010). Validation of the 2009 TNM version in a large multi-institutional cohort of patients treated for renal cell carcinoma: are further improvements needed?. Eur Urol.

[17] Choudhary S, Rajesh A, Mayer NJ, Mulcahy KA, Haroon A (2009). Renal oncocytoma: CT features cannot reliably distinguish oncocytoma from other renal neoplasms. Clin Radiol.

[18] Bechtold RE, Zagoria RJ (1997). Imaging approach to staging of renal cell carcinoma. Urol Clin North Am.

[19] Marshall ME, Pearson T, Simpson W, Butler K, McRoberts W (1990). Low incidence of asymptomatic brain metastases in patients with renal cell carcinoma. Urology.

[20] Zisman A, Pantuck AJ, Dorey F, Said JW, Shvarts O, Quintana D (2001). Improved prognostication of renal cell carcinoma using an integrated staging system. J Clin Oncol.

[21] Frank I, Blute ML, Cheville JC, Lohse CM, Weaver AL, Zincke H (2002). An outcome prediction model for patients with clear cell renal cell carcinoma treated with radical nephrectomy based on tumor stage, size, grade and necrosis: the SSIGN score. J Urol.

[22] Karakiewicz PI, Briganti A, Chun FK, Trinh QD, Perrotte P, Ficarra V (2007). Multi-institutional validation of a new renal cancer-specific survival nomogram. J Clin Oncol.

[23] Heng DY, Xie W, Regan MM, Warren MA, Golshayan AR, Sahi C (2009). Prognostic factors for overall survival in patients with metastatic renal cell carcinoma treated with vascular endothelial growth factor-targeted agents: results from a large, multicenter study. J Clin Oncol.

[24] Motzer RJ, Bacik J, Murphy BA, Russo P, Mazumdar M (2002). Interferon-alfa as a comparative treatment for clinical trials of new therapies against advanced renal cell carcinoma. J Clin Oncol.

[25] Thompson RH, Boorjian SA, Lohse CM, Leibovich BC, Kwon ED, Cheville JC, et al. Radical nephrectomy for pT1a renal masses may be associated with decreased overall survival compared with partial nephrectomy. J Urol. 2008;179(2):468–71 (discussion 72–3). 10.1016/j.juro.2007.09.077 18076931

[26] Simmons MN, Weight CJ, Gill IS (2009). Laparoscopic radical versus partial nephrectomy for tumors >4 cm: intermediate-term oncologic and functional outcomes. Urology.

[27] Hemal AK, Kumar A, Kumar R, Wadhwa P, Seth A, Gupta NP (2007). Laparoscopic versus open radical nephrectomy for large renal tumors: a long-term prospective comparison. J Urol.

[28] Lane BR, Tiong HY, Campbell SC, Fergany AF, Weight CJ, Larson BT, et al. Management of the adrenal gland during partial nephrectomy. J Urol. 2009;181(6):2430–6 (discussion 6–7). 10.1016/j.juro.2009.02.027 19371896

[29] Blom JH, van Poppel H, Maréchal JM, Jacqmin D, Schröder FH, de Prijck L (2009). Radical nephrectomy with and without lymph-node dissection: final results of European Organization for Research and Treatment of Cancer (EORTC) randomized phase 3 trial 30881. Eur Urol.

[30] Lane BR, Abouassaly R, Gao T, Weight CJ, Hernandez AV, Larson BT (2010). Active treatment of localized renal tumors may not impact overall survival in patients aged 75 years or older. Cancer.

[31] Karam JA, Rini BI, Varella L, Garcia JA, Dreicer R, Choueiri TK (2011). Metastasectomy after targeted therapy in patients with advanced renal cell carcinoma. J Urol.

[32] Motzer RJ, Hutson TE, Tomczak P, Michaelson MD, Bukowski RM, Rixe O (2007). Sunitinib versus interferon alfa in metastatic renal-cell carcinoma. N Engl J Med.

[33] Escudier B, Pluzanska A, Koralewski P, Ravaud A, Bracarda S, Szczylik C (2007). Bevacizumab plus interferon alfa-2a for treatment of metastatic renal cell carcinoma: a randomised, double-blind phase III trial. Lancet.

[34] Rini BI, Halabi S, Rosenberg JE, Stadler WM, Vaena DA, Ou SS (2008). Bevacizumab plus interferon alfa compared with interferon alfa monotherapy in patients with metastatic renal cell carcinoma: CALGB 90206. J Clin Oncol.

[35] Sternberg CN, Davis ID, Mardiak J, Szczylik C, Lee E, Wagstaff J (2010). Pazopanib in locally advanced or metastatic renal cell carcinoma: results of a randomized phase III trial. J Clin Oncol.

[36] Hudes G, Carducci M, Tomczak P, Dutcher J, Figlin R, Kapoor A (2007). Temsirolimus, interferon alfa, or both for advanced renal-cell carcinoma. N Engl J Med.

[37] Motzer RJ, Hutson TE, Cella D, Reeves J, Hawkins R, Guo J (2013). Pazopanib versus sunitinib in metastatic renal-cell carcinoma. N Engl J Med.

[38] Motzer RJ, Nosov D, Eisen T, Bondarenko I, Lesovoy V, Lipatov O (2013). Tivozanib versus sorafenib as initial targeted therapy for patients with metastatic renal cell carcinoma: results from a phase III trial. J Clin Oncol.

[39] Hutson TE, Lesovoy V, Al-Shukri S, Stus VP, Lipatov ON, Bair AH (2013). Axitinib versus sorafenib as first-line therapy in patients with metastatic renal-cell carcinoma: a randomised open-label phase 3 trial. Lancet Oncol.

[40] Escudier B, Eisen T, Stadler WM, Szczylik C, Oudard S, Siebels M (2007). Sorafenib in advanced clear-cell renal-cell carcinoma. N Engl J Med.

[41] Rini BI, Escudier B, Tomczak P, Kaprin A, Szczylik C, Hutson TE (2011). Comparative effectiveness of axitinib versus sorafenib in advanced renal cell carcinoma (AXIS): a randomised phase 3 trial. Lancet.

[42] Motzer RJ, Escudier B, Oudard S, Hutson TE, Porta C, Bracarda S (2008). Efficacy of everolimus in advanced renal cell carcinoma: a double-blind, randomised, placebo-controlled phase III trial. Lancet.

[43] Hutson TE, Escudier B, Esteban E, Bjarnason GA, Lim HY, Pittman KB (2014). Randomized phase III trial of temsirolimus versus sorafenib as second-line therapy after sunitinib in patients with metastatic renal cell carcinoma. J Clin Oncol.

[44] Bellmunt J, Pons F, Foreshew A, Fay AP, Powles T, Porta C, et al. Sequential targeted therapy after pazopanib therapy in patients with metastatic renal cell cancer: efficacy and toxicity. Clin Genitourin Cancer. 2014;12(4):262–9. 10.1016/j.clgc.2014.03.002 24795159

[45] Calvo E, Grünwald V, Bellmunt J (2014). Controversies in renal cell carcinoma: treatment choice after progression on vascular endothelial growth factor-targeted therapy. Eur J Cancer.

[46] Vera-Badillo FE, Templeton AJ, Duran I, Ocana A, de Gouveia P, Aneja P, et al. Systemic therapy for non-clear cell renal cell carcinomas: a systematic review and meta-analysis. Eur Urol. 2014. doi:10.1016/j.eururo.2014.05.010. 10.1016/j.eururo.2014.05.010 24882670

[47] Thian Y, Gutzeit A, Koh DM, Fisher R, Lote H, Larkin J, et al. Revised Choi imaging criteria correlate with clinical outcomes in patients with metastatic renal cell carcinoma treated with sunitinib. Radiology. 2014:132702. 10.1148/radiol.14132702 24869795

[48] León L, García-Figueras R, Suárez C, Arjonilla A, Puente J, Vargas B (2014). Recommendations for the clinical and radiological evaluation of response to treatment in metastatic renal cell cancer. Target Oncol..

